# Book reviews

**DOI:** 10.1155/S1463924691000305

**Published:** 1991


### Book reviews

**Computerized Quality Control: Programs for the Analytical Laboratory,** 2nd edition
By T. F. Hartley (Ellis Horwood, Chichester, 1991). £29.95. ISBN 0-113-151614-0.

This book, in the fine tradition of Ellis Horwood publications, has been updated considerably and now fully fits the needs of the analytical market. The pressure on all laboratories to achieve national and international accreditation and to achieve good laboratory practice has placed a demand on quality-control procedures. This book, written from practical experience, allows any laboratory, with just the addition of an IBM PC, to introduce good quality-control procedures. The necessary software is available from the publisher on disk.

The first edition was extremely well received; to meet new demands in legislation a chapter has been added to the second edition to deal with external quality-control schemes. More attention has also been given to identifying the trends in quality-control data. Specifically, the auto-regressive moving average model to identify trends is introduced in this text. The book provides the user with all that is required and identifies possible errors introduced by computational errors in the PC. The user does not have to be frightened by statistical approaches and can adequately deal with the quality assurance problems of analytical chemistry without passing them on to an external statistician.

The book includes 70 references and identifies other areas of suitable reading. It is attractively produced with the distinctive cover styles of the Ellis Horwood publishing house. An extremely valuable addition to the analytical chemistry laboratory and good value at £29.95.

**On-Line Coupled LC-LG**
By Konrad Grob (Hüthig Buch Verlag GmbH, Heidelberg, Germany, 1991). 462 pp. DM 178. ISBN 3-7785-1872-0.

This manual, which is part of a series on chromatographic methods, concentrates on the techniques for transferring the usually large volumes of LC eluent to GC. Selective solvent evaporation at a sufficiently high rate is at the heart of the transfer. The LC-GC interfaces required for the different transfer techniques are described in detail, followed by a discussion of applications in order to show the practical implications.

The book is based on an inductive approach. After a presentation of partial transfer from LC to GC (Section 2), considered to be of limited interest, Section 3 describes the basic concepts that it should be simple to rebuild the systems. Section 6, on LC techniques suitable for LC-GC, shows how much is left to be done in this field. Sections 7 and 9 describe the presently used transfer techniques (retention gap techniques, concurrent eluent evaporation), while Sections 8 and 10 discuss applications involving the corresponding transfer methods – they should illustrate theory through practice. Section 11 gives some comments on the idea of using liquefied gases on supercritical fluids for sample preparation instead of ordinary LC eluents.

As coupled techniques receive more attention a basic reference text like this forms an ideal foundation for the researcher.

### International Symposium on Laboratory Automation and Robotics

The ninth International Symposium on Laboratory Automation and Robotics will be held from 20 to 23 October 1991 at the Boston Park Plaza Hotel and Towers. Using the theme, 'Laboratory Automation – Fad of the 1980s or Necessity of the 1990s', this year's Symposium will focus on recent developments that provide state-of-the-art automation for today's chemists.

Two keynote speakers from the pharmaceutical industry will be featured in the opening session on Monday morning. David B. Weinstein, Director of the Lipid and Lipoprotein Metabolism Department at Sandoz Research Institute will speak on 'Jumping into the twentieth century before it's too late: is laboratory robotics still in its infancy?' Bernd C. Schade, Director of Quality Assurance for the Consumer Healthcare Division of Miles Inc., will discuss 'Quality control in the year 2000'. Additionally, several laboratory managers will provide their perspective on implementing laboratory automation.

The three-day programme will include over 75 podium and poster presentations describing laboratory robotics and laboratory workstation procedures from pharmaceutical, biotechnology, environmental, industrial hygiene, and chemical/petrochemical laboratories. The Symposium's technical presentations and informal discussions provide an excellent opportunity for laboratory automation users and novices to exchange ideas and applications experience. Session topics include:

- Microplate assays.
- Environmental analysis.
- Managing laboratory automation.
- Chromatography sample preparation.
- Biology and biotechnology.
- Pharmaceutical analysis.
- Intelligent automation.
- Advanced technology.

*For more information contact: Christine O'Neil, Zymark Corporation, Zymark Center, Hopkinton, Massachusetts 01748, USA. Tel.: 508 435 9500 ext 2224; fax 508 435 3439.*

